# Identification of Putative Elicitors From Plant Root Exudates Responsible for PsoR Activation in Plant-Beneficial *Pseudomonas* spp. by Docking and Molecular Dynamics Simulation Approaches to Decipher Plant–Microbe Interaction

**DOI:** 10.3389/fpls.2022.875494

**Published:** 2022-04-06

**Authors:** Diksha Sati, Tushar Joshi, Satish Chandra Pandey, Veni Pande, Shalini Mathpal, Subhash Chandra, Mukesh Samant

**Affiliations:** ^1^Cell and Molecular Biology Laboratory, Department of Zoology (DST-FIST Sponsored), Soban Singh Jeena University Campus, Almora, India; ^2^Department of Zoology, Kumaun University, Nainital, India; ^3^Department of Biotechnology, Kumaun University, Sir J C Bose Technical Campus, Bhimtal, India; ^4^Computational Biology & Biotechnology Laboratory, Department of Botany, Soban Singh Jeena University Campus, Almora, India

**Keywords:** inter-kingdom signaling, saponarin, 2-benzoxazolinone, molecular docking, molecular dynamic simulation

## Abstract

Plants and rhizobacteria are coexisting since the beginning, but the exact mechanism of communication between them remains enigmatic. The PsoR protein of plant-beneficial *Pseudomonas* spp., a group of root-associated bacteria, is known to produce a range of antifungal and insecticidal secondary metabolites like 2,4-diacetyl phloroglucinol (DAPG), pyrrolnitrin, and chitinase making them great biocontrol agents and thus helping in plant growth promotion. To better understand the inter-kingdom signaling between plants and plant growth-promoting rhizobacteria (PGPR), the interaction of PsoR with various root exudates was investigated computationally. For this, we first modeled the PsoR protein and confirmed it using the Ramachandran plot. A total of 59 different low molecular weight phytochemicals, secreted as root exudates by plants, were identified by extensive text mining. They were virtually screened with the PsoR protein by molecular docking. Based on the lowest binding energy, ranging from −7.1 to −6.3 kcal mol^−1^, the top five exudates were chosen. To analyze the stability of the docked protein–ligand complex, a molecular dynamics (MD) simulation of 100 nanoseconds was done. Two root exudates, saponarin and 2-benzoxazolinone (BOA), showed suitable binding with PsoR by forming hydrogen, hydrophobic, and Van der Waals interactions. To confirm the MD simulation results, RMSF, RG, SASA, and interaction energy were calculated. This computational study first time reports that saponarin and 2-BOA, predominantly present in the root exudates of barley and wheat, respectively, demonstrate effective binding with the modeled PsoR protein and are likely of showing cross-kingdom interactions.

## Introduction

**Figure 1:**
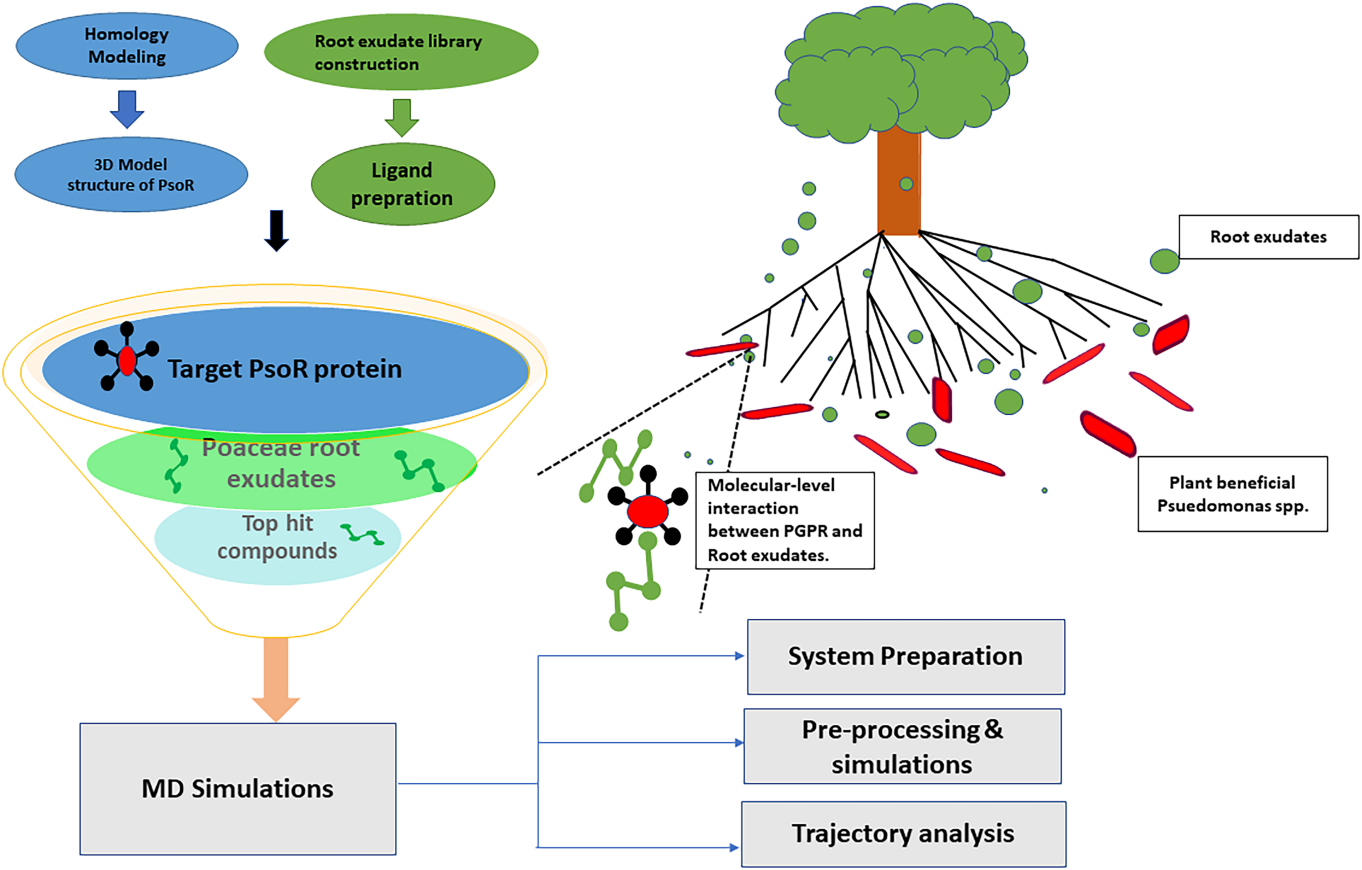
Schematic representation of various steps employed in the present study.

Plants and rhizobacteria have been living nearby and co-evolving for a considerably long period, during which time both have been subjected to the signaling molecules formed and released by the other (Lyu et al., 2021). Rhizobacteria employ various direct and indirect mechanisms of growth promotion in their host plants (Papenfort and Bassler, 2016). Plants, in return, exude some active metabolites from their roots, of diverse chemical nature, and help in shaping microbial communities in the rhizosphere (Papenfort and Bassler, 2016; Sasse et al., 2018). Plant–microbe interaction not only maintains plant productivity but also ensures global food security by supporting a healthy ecosystem (Sati et al., 2022). Traditionally, LuxR protein has been known as an important regulator of quorum sensing (QS) signaling, by detecting the N-acyl homoserine lactones (AHLs) molecules in a cell density-dependent manner and regulating the target gene (Papenfort and Bassler, 2016). LuxR solos are present in various bacteria and play diverse roles like plant growth promotion, nodule formation, locomotion, extra-chromosomal DNA transfer, pathogenesis, and regulation of QS (Subramoni and Venturi, 2009). Moreover, LuxR solo of plant-associated bacteria (both plant-friendly and plant-pathogenic ones) differs in only one or two critical amino acid residues in the auto-inducer domain, which possibly could allow them to interact with plant-derived signal molecules instead of AHLs, hence facilitating inter-kingdom signaling (Subramoni et al., 2011; González et al., 2013). Various LuxR solo proteins have been reported, *viz.* XccR in *Xanthomonas campestris*, OryR in *Xanthomonas oryzae*, NesR in *Sinorhizobium meliloti*, and PsoR in plant-beneficial *Pseudomonas* spp. The PsoR protein of *Pseudomonas fluorescens* species complex is concerned with the regulation of biocontrol traits. The *psoR* gene codes for a 252-residue long protein containing an N terminal inducer-binding domain (PF03472) and a C terminal DNA-binding domain (PF00196), a characteristic of LuxR proteins involved in QS. PsoR directs transcriptional modulation of various antimicrobial-related genes in plant-beneficial *Pseudomonas* spp. in reaction to plant compounds (Patel et al., 2013). Pyrrolnitrin biosynthetic genes, DAPG genes, and genes involving synthesis of chitin-binding protein and chitinase were upregulated by PsoR (Haas and Keel, 2003). The interconnection between the PsoR protein of plant-beneficial *Pseudomonas* spp. and root exudates of rice and wheat plants has been previously reported (González et al., 2013). Further, the *psoR* gene in *P. fluorescens* CHA0 protects the wheat plant against *Pythium ultimum* infection because, when the *psoR* gene was knocked out from *P. fluorescens*, it was unable to protect the plant root, and a 30% decrease in fresh weight of root was seen in comparison with control. When this experiment was conducted taking cucumber as the host plant, though *P. fluorescens* CHA0 imparted protection from the pathogen, there was no considerable difference between the wild-type strain and the *psoR* mutant (Subramoni et al., 2011). These findings suggest that the *psoR* gene is likely specific for a molecule present in Poaceae plants. Tracing the given lines of evidence, we designed an *in silico* study to determine the potential plant root exudates interacting with PsoR and further manifesting its role in plant growth promotion by biocontrol. By cutting time and expenditure and hastening studies on desired interactions with low molecular weight molecules, computational approaches have been highly useful tools in finding the function of any protein. Molecular docking has shown to be one of the most useful *in silico* strategies for identifying new bioactive compounds from large chemical libraries. During docking, the primary binding mode for each small molecule is analyzed inside a target binding pocket, and a docking score is assigned to them based on their binding propensity. It can also be used to assist lead refinement by showing how a certain hit can be altered to optimize protein–ligand interaction. In comparison to *in vitro* techniques, molecular docking is both cost-friendly and time-saving. However, many estimations are generated by docking procedures and majority of them absent receptor flexibility. As a result, the dependability of the resulting protein–ligand complexes is limited. Consequently, molecular docking must be paired with other *in silico* methods to obtain more accurate results. Combining docking with MD simulations is a prevalent approach to strengthen results. MD simulations work by improving the topologies of the already docked receptor–ligand complexes, providing more accurate interaction energies, and revealing the ligand-binding mechanism. Molecular modeling, docking, and dynamics simulation studies have been immensely helpful in the past for elucidating the structure and function of many plant proteins. For example, computational analysis of α-expansin protein from mountain papaya fruit was made possible using a comparative modeling strategy and its optimization by MD simulations (Gaete-Eastman et al., 2015). Similarly, structural insights of rice protein urease were provided by using homology modeling and MD simulation approach (Kumar et al., 2018). However, computational studies made on plant–microbe interaction are quite limited. Earlier, the ligand-binding sites of QS LuxRs were mapped using structure-based sequence alignment and structural superimposition (Covaceuszach et al., 2013). Bioinformatics and molecular docking-based approaches have previously been successfully applied for providing structural knowledge of regulatory domains of many Lux R family proteins, including PsoR (Karplus and McCammon, 2002; Trott and Olson, 2010; Laskowski et al., 2018). However, more focused docking and simulation-based studies were required to figure out the exudate compounds eliciting bacterial protein PsoR. Hence, in our study, a total of 59 diverse plant root exudate compounds were taken and those showing strong interaction with PsoR were selected by the computational screening method (Figure 1). Using an *in silico* study, we found two putative root exudates, *viz.* saponarin and 2-benzoxazolinone (BOA), positively interact with PsoR of *P. fluorescens*. Saponarin is the major flavonoid found in barley, while BOA is the major secondary metabolite of wheat, maize, and rye. We also explored PsoR catalytic sites and using molecular dynamics (MD) simulation authenticated the reliability of PsoR-exudate complexes.

## Materials and Methods

### Molecular Modeling

**Figure 2:**
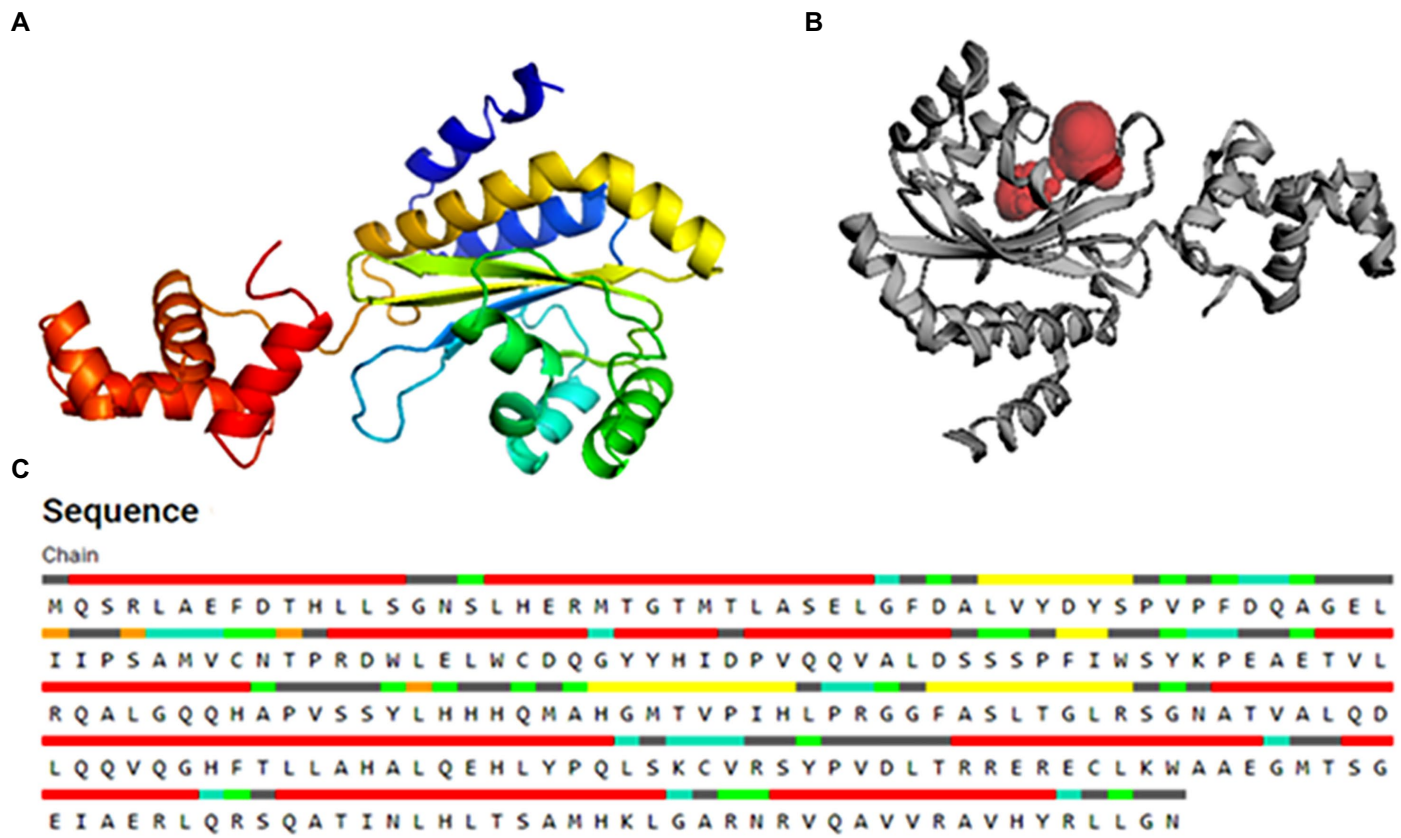
The 3D structure of the PsoR protein generated by the “Easy Modeller” homology modeling tool **(A)**, active binding site in protein **(B)**, and residues participating in active binding **(C)**.

Since the three-dimensional (3D) structure of PsoR of *Pseudomonas* is not available so far, we first procured the sequence of PsoR of *Pseudomonas protegens* Pf-5 from NCBI (accession number–WP_011063531.1), and using the “Easy Modeller” homology modeling tool, we later generated the 3D structure of the protein (Figure 2A; Kuntal et al., 2010). For the additional refinement of the artificially constructed model of PsoR, it was subjected to energy minimization, side-chain refinement, loop refinement, etc. To authenticate the refined structure, it was uploaded into the PDBsum server (Laskowski et al., 2018).

### Active Site Analysis

For verifying the active sites within the modeled PsoR protein and for tracing, characterizing, and quantifying its spatial attributes, the CASTp webserver was employed (Lozano et al., 2012). Various catalytically active amino acid residues were identified with the help of CASTp 3.0, which might be involved in protein–ligand interaction. For rigorous docking, the estimated pocket attached with the amino acid residues linked with the reference molecule was taken into account.

### Ligand Preparation

For the selection of the low molecular, root exudates of Poaceae, we did an extensive literature survey on root exudates of barley, rice, rye, maize, and wheat plants. We created a phytochemical library of total of 59 diverse plant root exudates to be used in our study. The 3D structures of the root exudates of Poaceae and the reference molecule in the SDF format were retrieved from NCBI Pubchem.^1^ Using Open Babel software (Version 2.3.1), all root exudates and the reference molecule in the SDF files were translated to PDB format.

### Molecular Docking and Visualization

By employing PyRx open-source software, molecular docking was carried out to generate a set of potential ligand topologies and conformations at the binding site (GUI version 0.8 of AutodockVina; Trott and Olson, 2010). The calculation of the bound configurations by the PyRx software relies upon the binding affinity. For molecular docking studies, the grid center was set to *X* = 10.84, *Y* = −24.12, and *Z* = 13.30, and the dimensions of the grid box were set as 25.00, 25.30, and 25.96 Å with a spacing of 0.375 Å between the grids points. The number of exhaustiveness was set to eight by default. The polar hydrogen atoms and Kollamen charges were assigned during receptor and ligand preparation. Compounds were computationally screened with rigorous molecular docking in the catalytic center of the PsoR protein while allowing ligand molecules to be flexible. Total nine bound conformations of each ligand were generated using this program based on binding affinity. For further investigation, only the lowest binding energy conformational states of the ligand molecules were chosen. The Ligplotv.1.4.5 program was used to visualize and examine the molecular characteristics among protein–ligand complexes, such as hydrogen bonds and bond lengths (Karplus and McCammon, 2002). However, the 3-D protein–ligand complex was studied using PyMol molecular visualization tool (Pronk et al., 2013).

### Molecular Dynamics Simulations

Molecular dynamics simulations were used to determine the structural stability of the protein–ligand complex under varying physiological circumstances (Karplus and McCammon, 2002). MD simulations were used to test the top compounds found by molecular docking. GROMACS was employed to conduct all of the MD simulations (Jorgensen et al., 1983). To generate topologies for the protein–ligand complex, the CHARMM 36 force field was used (Kumari et al., 2014). In the topology of the protein–ligand complex, the entire bonded and nonbonded factors were described, e.g., valency, atom groups, and bond connectivity. To mimic the biological environment, molecules have been dissolved in water. The water model of TIP3P was then used to construct a water solvated system with dodecahedral periodic boundary conditions and box vectors of equal length 9.81 nm. The solutes were placed in the simulation box with a minimum distance to the box edge of 10 Å (1.0 nm). The addition of three Na^+^ ions neutralized each solvated system. After adding the ions, we undertook energy minimization on the protein–ligand complex to confirm that the scaffold had no steric clashes and an appropriate starting structure. This stage was processed at 10 kJ/mol using the steepest descent algorithm and the Verlet cutoff method. Furthermore, the equilibration of the protein–ligand complex was accomplished in two stages. Equilibration was carried out at 300 K for 100 ps under the NVT ensemble. It kept the system’s temperature in balance. The NPT ensemble used Parrinello–Rahman simulation to perform second-phase equilibration. Here also the system was treated with a constant temperature (300 K) and pressure (1 atm) with a 2 fs time phase. The MD simulations of the protein–ligand were carried out for 100 ns. The root-mean-square divergence (RMSD), the radius of gyration (RG), root-mean-square fluctuation (RMSF), hydrogen bonds, solvent accessible surface area (SASA), and principal component analysis (PCA) were quantified to analyze the stability of the enzyme and protein–ligand complex. With the help of hydrogen bond analysis, the total number of unique hydrogen bonds generated during the protein–ligand reaction in the MD simulation was estimated.

### Binding Free Energy Calculation Using MM-PBSA

The MMPBSA (molecular dynamics Poisson–Boltzmann surface area) method is commonly used to determine the binding free energy and predict the stability of the protein–ligand complex following MD simulation (Kumari et al., 2014). Following that, using the molecular mechanics Poisson–Boltzmann surface area (MM-PBSA) procedure provided in the g_mmpbsa package, an extensive analysis of the binding free energy (DGbind) was done (Kuzmanic and Zagrovic, 2010). For the calculation of binding free energy of protein–ligand complexes, both free solvation energy (polar and nonpolar solvation energies) and potential energy (electrostatic and Van der Waals interactions) are required, which were measured by the MMPBSA approach. We evaluated the nonbonded interaction energy between protein and ligands after MD simulation was completed, using conditions analogous to MD simulation for computing the extent of the interaction between protein complexes.

## Results

In the current study, we docked the modeled PsoR protein with root exudates of Poaceae and investigated the interactions undertaking between PsoR-saponarin and PsoR-BOA at the molecular level using MD simulations.

### Homology Modeling

**Figure 3:**
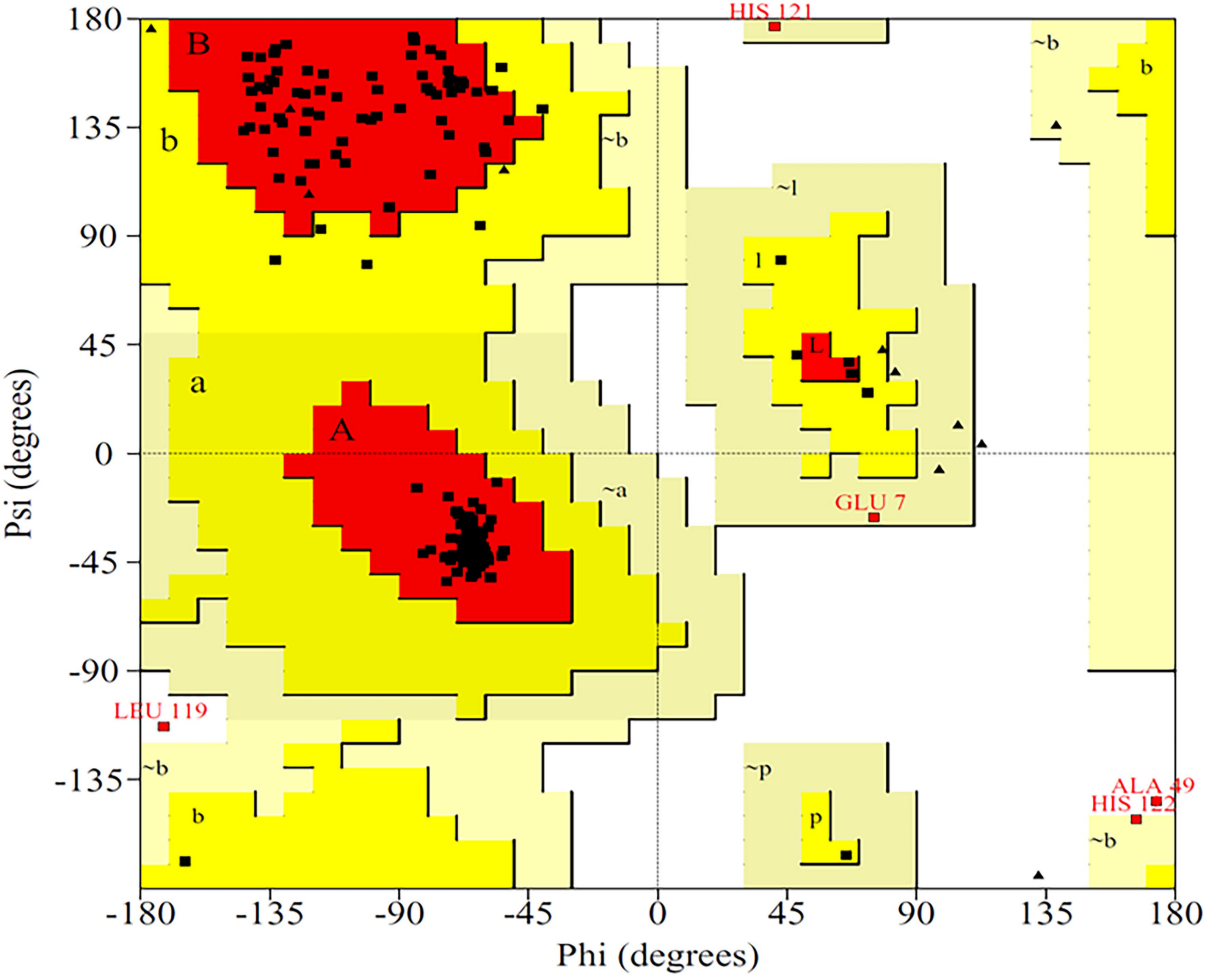
Ramachandran plot for validating the 3D structure of PsoR of *Pseudomonas protegens* Pf-5.

The primary structure of the PsoR protein of *P. protegens* Pf-5 was retrieved from NCBI, and BLASTP was used to recognize its cognate templates from the Protein Data Bank (PDB) database. The PsoR query is comparable to the crystal structure of PsoR in plant-beneficial *Pseudomonas* spp., according to BLASTP findings (PDB ID 6MWZ, 3SZT, and 4Y17), with 29.65, 29.08, and 27.15% identity and 88, 76, and 84% query coverage, consecutively. PDB ID 6MWZ corresponds to QS receptor protein, LasR of *Pseudomonas aeruginosa* UCBPP-PA14. Similarly, PDB ID 3SZT corresponds to QS control repressor, QscR of *P. aeruginosa* ATCC 15692, whereas PDB ID4Y17 is of a QS transcriptional regulator, SdiA, found in *Escherichia coli* K12. Later, Easy Modeller software was put to use for predicting the 3D structure of PsoR by considering these three structures as templates. Additionally, the Swiss-Pdb Viewer program (Guex and Peitsch, 1997) was used to minimize the energy of the predicted structure. The accurateness of the predicted structure is then authenticated using the ProQ (Wallner and Elofsson, 2003) and ProSA-web (Wiederstein and Sippl, 2007) servers. The quality of the projected model was analyzed using LG and MaxSub scores, which were estimated to be about 9.991 and −0.605, respectively, placing the model in the good prediction area. Likewise, the ProSA-web server presents the *Z*-score as a criterion to determine the total value of the PsoR protein, having a magnitude of −7.56. This suggests that the 3D structure has been accurately predicted. The 3D model of protein also demonstrated that about 92.8% of the total residues fall within the permitted region of the Ramachandran plot (Figure 3), further confirming the stereochemical stability of the PsoR homology template.

### Active Site Confirmation

The substrate accessible pockets and catalytic sites on the putative PsoR protein were analyzed using the Computed Atlas of Surface Topography of Proteins (CASTp) algorithm. Following are the amino acid residues in the pocket, which were selected for virtual screening: Val38, Asp40, Ser42, Pro43, Ala49, Gly50, Glu51, Leu52, Ile54, Leu67, Trp70, Tyr76, His77, Asp79, Val81, Gln82, Ala85, Leu86, His120, His121, Thr129, Phr139, Ser141, and Ser143 (Figure 2C). PsoR has an active site area of 184.972 and a volume of 118.953, according to CASTp findings (Figure 2B).

4-[3-(methylsulfonyl)phenoxy]-N-[(1S,3S,5S)-2-oxobicyclo[3.1.0]hexan-3-yl]butanamide (K5M) was taken as a reference compound as it was co-crystallized with PDB ID-6MWZ because this protein showed highest query cover with PsoR protein.

### Molecular Docking

**Figure 4:**
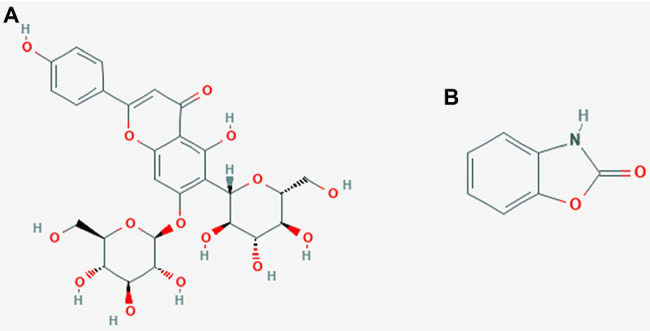
2D structures of saponarin **(A)**, and BOA **(B)** used in the molecular docking and MD simulation study.

**Figure 5:**
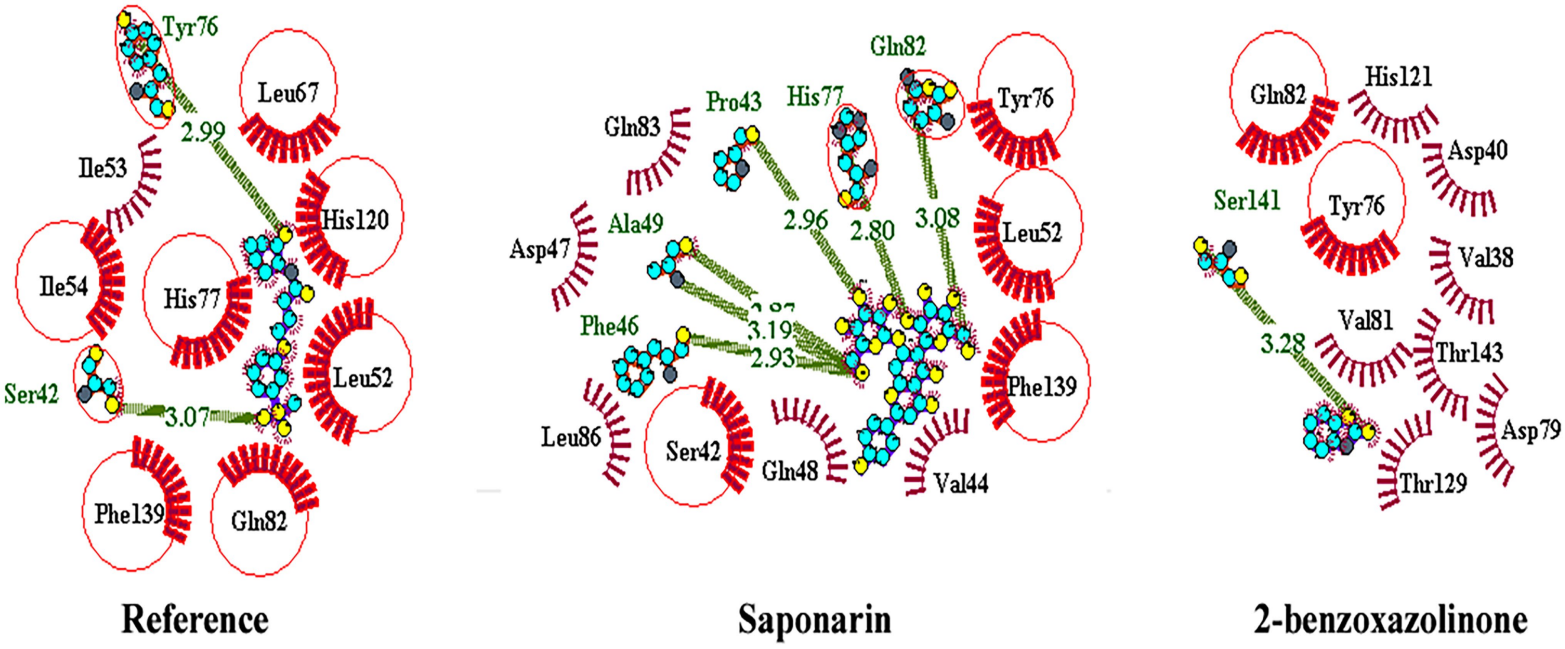
2D interaction of protein–ligand with H-bonds and hydrophobic bonds between top hit ligands (saponarin and BOA) and PsoR. (Dotted green lines denote hydrogen bonds, red ignited arcs indicate hydrophobic interactions, and red circle and red ellipses reflect common residues with reference.)

About 59 substrates were enriched by docking with the PsoR receptor (Supplementary Table 1). Among the total 59 root exudates, the best five were chosen from molecular docking, having better or nearly equal binding energy in comparison to the reference molecule K5M (−7.0 kcal mol^−1^; Table 1), and only those five exudates were further subjected to MD simulations. The MD simulations revealed that out of five, only two root exudates, i.e., saponarin and 2-benzoxazolinone (BOA), showed suitable binding with PsoR protein. Hence, we have targeted only these two ligands, i.e., saponarin and 2-benzoxazolinone (BOA) in a later study (Figure 4). The molecular docking study of PsoR with saponarin exposed that the catalytic site of the PsoR protein is made up of 5 amino acid residues, *viz.* Pro43, Phe46, Ala49, His77, and Gln82 amino acids, which are stabilized by five hydrogen bonds, whereas Ser42, Val44, Asp47, Gln48, Leu52, Tyr76, Gln83, Leu86, and Phe139 take part in hydrophobic interaction. Likewise, BOA exhibited association with Ser141 by one H-bond, although Val38, Asp40, Tyr76, Asp79, Val81, Gln82, His121, Thr129, and Thr143 residues were linked to PsoR through hydrophobic interaction (Figure 5). The results imply that the tested compounds may act similarly to the reference molecule K5M.

**Table 1:** Molecular docking scores of various screened root exudates with PsoR of *Pseudomonas protegens* Pf-5.

S. no.	Name of the substrate	Compound ID	Molecular formula	Binding energy, kcal mol^−1^
1	Reference (K5M)	137,628,326	C_17_H_21_NO_5_S	−7.0
2	Saponarin	441,381	C_27_H_30_O_15_	−7.1
3	o-coumaric acid	637,540	C_9_H_8_O_3_	−6.6
4	2-Benzoxazolinone	6,043	C_7_H_5_NO_2_	−6.4
5	trans-cinnamic acid	444,539	C_9_H_8_O_2_	−6.4
6	p-hydroxybenzoic acid	135	C_7_H_6_O_3_	−6.3
7	Adenosine	60,961	C_10_H_13_N_5_O_4_	−6.2
8	Phthalic acid	1,017	C_8_H_6_O_4_	−5.8
9	Ferulic acid	445,858	C_10_H_10_O_4_	−5.6
10	D-maltose	6,255	C_12_H_22_O_11_	−5.5

### Molecular Dynamic Simulation

Molecular dynamics simulation was used to assess the stability of the PsoR-exudate complex. It was used to study the physical mobility of atoms and molecules, as well as to predict conformational changes at the molecular level. As the binding model of BOA and saponarin was shown to be suitable based on PyRx’s molecular docking performance, the structural alterations of only these two exudates were further examined in this study. The binding stability and free energy of protein–ligand complexes were determined using a molecular dynamics simulation with a time step of 100 ns. The protein–ligand complex in PsoR activation and its effects in the simulation was studied using RMSD, RMSF, RG, SASA, and MMPBSA measurements. As already mentioned, according to the MD simulation data, only two exudates were found to be stable against the PsoR enzyme (Figure 4). Consequently, in the following study, only the effects of those two exudates, saponarin and BOA, were reported.

#### RMSD of the Protein–Ligand Complex

**Figure 6:**
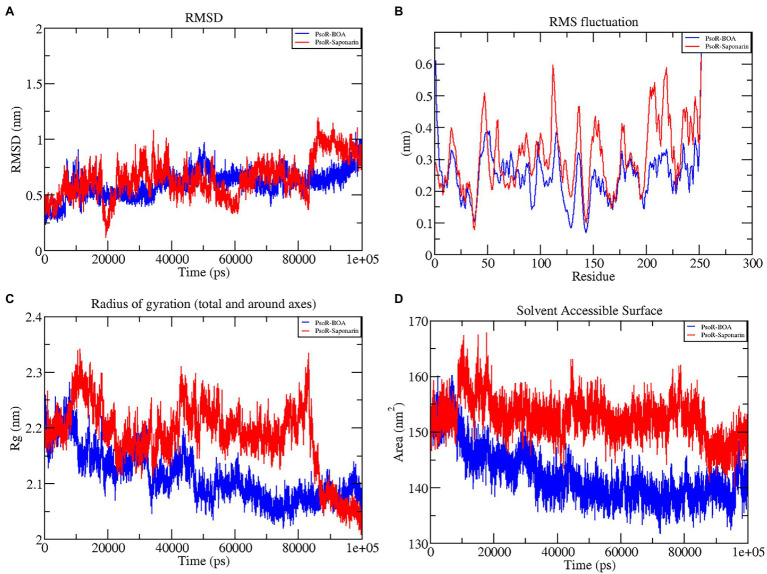
Graphical representation of RMSD **(A)**, RMSF **(B)**, RG **(C)**, and SASA **(D)** profile of the protein–ligand complex for 100 ns MD simulation. The color code blue indicates PsoR-BOA, and red indicates PsoR-saponarin.

The variation in the protein–ligand complex was assessed using the root-mean-square deviation (RMSD) at 100 ns. The variations generated in a protein during simulation characterize its structural stability. The nature of proteins with fewer variations is more predictable. The RMSD value was computed for the α-carbon of saponarin and BOA with PsoR. Figure 6A shows the curve of RMSD (nm) vs. time (ps) for both ligands. The RMSD trajectory of the protein and all of the complexes attained equilibration, as shown in the diagram. For the complexes, PsoR-BOA and PsoR-saponarin, the average RMSD values were 0.59 ± 0.09 nm, and 0.65 ± 0.13 nm, respectively, (Table 2). RMSD is a measure in MD simulations to evaluate the equilibration and mobility of proteins/enzymes and even to estimate the distance between the protein’s backbone and atoms (Sargsyan et al., 2017). Both the protein–ligand complexes, namely PsoR-BOA and PsoR-saponarin, are stable, as evidenced by a small variation and a reduced RMSD. Furthermore, the results confirmed that the MD simulations for PsoR remained constant at 100 ns during the reaction which indicates that the PsoR enzyme can interact with saponarin and BOA ligands. To better understand the variation in the complex during biological processes, other investigators have also evaluated the RMSD for protein–ligand complexes (Joshi et al., 2021).

**Table 2:** The average values of RMSD, RG, SASA, and interaction energy of different protein–ligand complexes.

Protein–ligand complex	Average RMSD (nm)	Average RG (nm)	Average SASA (nm^2^)	Interaction energy (kJ/mol)
PsoR-2-benzoxazolinone	0.59 ± 0.09	1.8 ± 0.004	142.15 ± 3.7	−71.1813
PsoR-saponarin	0.65 ± 0.13	1.8 ± 0.006	152.62 ± 2.7	−167.032

#### RMSF of the Protein–Ligand Complex

To predict the movement of an atom’s position at a specific temperature and pressure for two sets of PsoR-exudate complexes, an RMSF evaluation was done. The RMSF findings depicted the flexible areas of the protein and estimated the overall variation of protein during the MD simulation. A lower RMSF reading is often considered good as it signifies that the protein–ligand combination was more stable, while a larger reading means MD was more flexible. For the protein–ligand complexes, variations in the component residue of the enzyme were observed over the 100 ns trajectory time (Figure 6B). For the duration of the encounter of the PsoR-exudate complex, a variation of less than 0.4–0.2 nm was detected in the active binding region, which is perfectly acceptable (Table 3). Therefore, as per the RMSF readings, it can be said that both the complexes were stable throughout MD simulations, with reduced fluctuation and increased stability. RMSF has been used to investigate protein–ligand complexes in the past. The findings demonstrate that when the flexibility of an enzyme is hindered, conformational rearrangements for substrate binding occur during catalysis.

**Table 3:** Root-mean-square fluctuation (RMSF) values (nm) of residues involved in the interaction of the protein–ligand complex.

Protein–ligand complex	Hydrogen bond interaction			Hydrophobic bond interaction		
	No. of bond	Residues involved	RMSF value	No. of bond	Residues involved	RMSF value
PsoR-2-benzoxazolinone	1	Ser141	0.11	9	Val38	0.10
					Asp40	0.15
					Tyr76	0.23
					Asp79	0.26
					Val81	0.20
					Gln82	0.21
					His121	0.18
					Thr129	0.08
					Thr143	0.06
PsoR-saponarin	5	Pro43	0.33	9	Ser42	0.27
		Phe46	0.46		Val44	0.40
		Ala49	0.40		Asp47	0.50
		His77	0.24		Gln48	0.47
		Gln82	0.32		Leu52	0.25
					Tyr76	0.21
					Gln83	0.35
					Leu86	0.36
					Phe139	0.29

#### Radius of Gyration

The radius of gyration indicates the degree of compactness of an enzyme–substrate complex. It deals with the folding and unfolding of proteins. The radius of gyration was estimated by utilizing the final 100 ns trajectories in this study. The mean RG readings for BOA and saponarin were 1.8 ± 0.004 and 1.8 ± 0.006 nm, correspondingly (Table 2). The steadiness in the RG reading indicates folding of the enzyme, whereas fluctuations in the RG reading correspond to the unfolding of enzymes (Lobanov et al., 2008). The RG results uncovered that every protein–ligand complex displayed an RG value that was comparatively close and in line with the expected statistics (Figure 6C). This indicates that both the exudate compounds were completely superimposed over PsoR protein which reflected in compaction and stabilization of the protein–ligand complex (Pande et al., 2021).

#### Solvent Accessible Surface Area

Solvent accessible surface area (SASA) is the abbreviation for solvent accessible surface area. For the duration of MD simulation, the total surface area of protein that can be admitted by the water solvent is evaluated by SASA and it can be used further for investigating communication within the solvent and the complex. For BOA, the mean SASA was found to be 142.15 ± 3.7 nm^2^, whereas for saponarin it was evaluated as 152.62 ± 2.7 nm^2^ (Table 2). Hence, in our findings the SASA values for the two aforementioned complexes remained astonishingly stable in the 100 ns MD simulation, implying that the structure of the protein has not changed considerably (Figure 6D). The PsoR complexes carried an elevated SASA reading in both cases. This may be due to the gradual unfolding of the critical amino acids in the active sites of PsoR protein, after interacting with BOA and saponarin. As a result, polar water molecules get entry into the newly opened pocket. This indicates that the channels and cavities of the proteins are essential in recognition of bacterial PsoR protein and its further binding with plant root exudates like BOA and saponarin. In conclusion, we have observed that PsoR’s surface has generated cavities.

The results of this study demonstrate that as a result of GROMACS’ charm force field electrostatic interaction, PsoR complexes develop spontaneously in an aqueous solution. These findings shed light on the interaction of BOA and saponarin within the enzyme cavity, and also on the electrostatic attraction aiding in stable binding.

#### Interaction Energy of Protein–Ligand Complex

**Figure 7:**
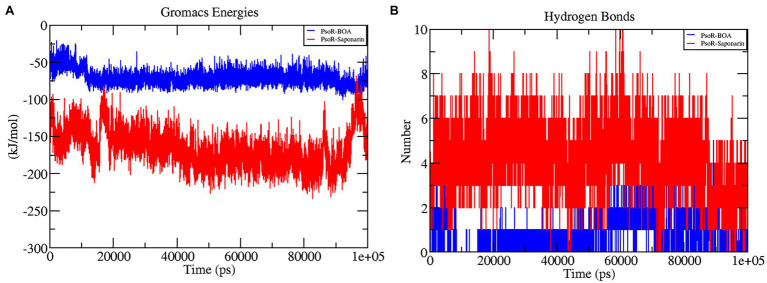
The graphs representing interaction energy **(A)**, and the number of H-bonds **(B)** as a function of time. The color code blue indicates PsoR-BOA, and red indicates PsoR-saponarin.

The interaction energy is used to analyze the efficacy of the protein–ligand combination. For confirmation of the strength of protein and ligand molecule, interaction energy was used. The interaction energy was calculated using GROMACS’ Parrinello–Rahman parameter. The estimated interaction energy values for the BOA and saponarin were −71.1813 kJ mol^−1^ and −167.032 kJ mol^−1^, respectively, in a 100 ns simulation session (Figure 7A; Table 2). The interaction energy results suggest that PsoR efficiently recognizes and binds with BOA and saponarin. As a consequence of these findings, an inter-kingdom signaling circuit between root exudates and plant-associated bacteria can be further explored.

#### Hydrogen Bonding Analysis of Protein–Ligand Complex

In the interaction of enzyme and substrate, hydrogen bonding is considered critical (Chen et al., 2016). It is required for the substrate’s selectivity, metabolism, and catalysis. The two complexes were explored further for a better understanding of the hydrogen bond in their association with PsoR protein. The total H bonds were recorded at the end of 100 ns. Saponarin contains 10 H bonds, whereas BOA has four H bonds, as per the findings (Figure 7B). These results indicate that saponarin binds to the PsoR active site more efficiently than BOA.

#### Principal Component Analysis

**Figure 8:**
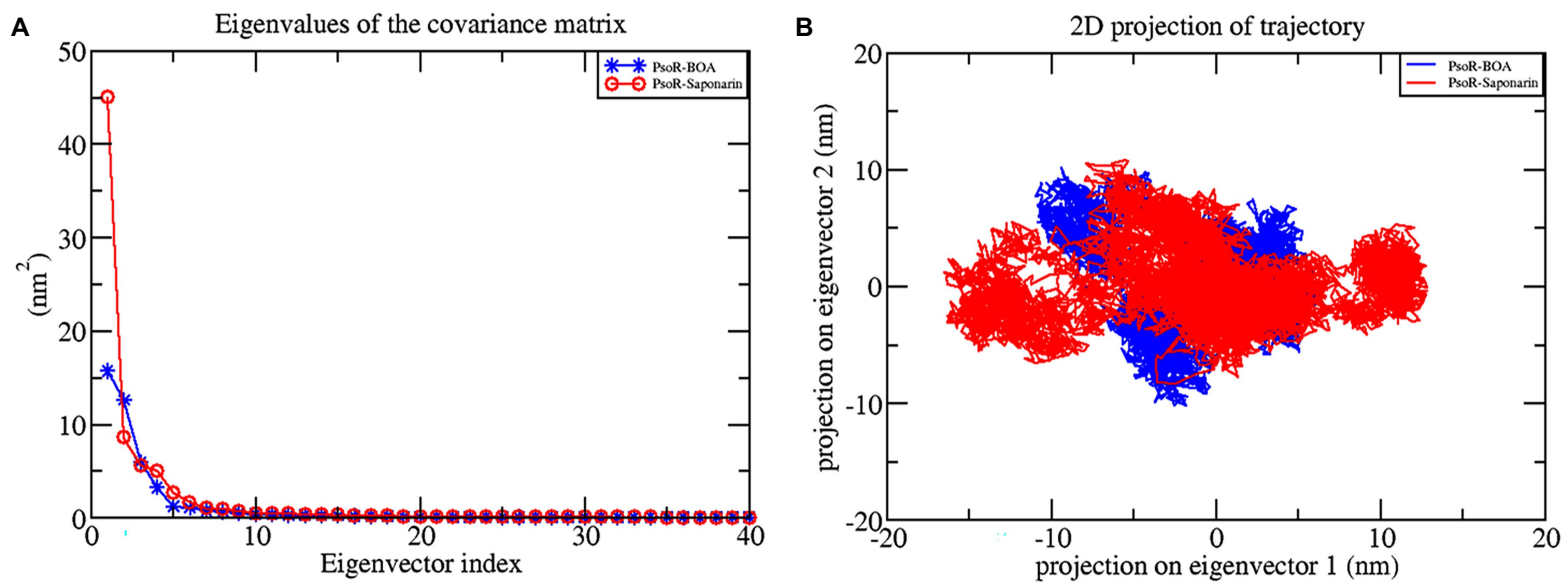
Principal component analysis: plot of the eigenvalues vs. first 40 eigenvectors **(A)**, the first two eigenvectors showing the protein motion in phase space for all the complexes **(B)**. The color code blue indicates PsoR-BOA, and red indicates PsoR-saponarin.

**Figure 9:**
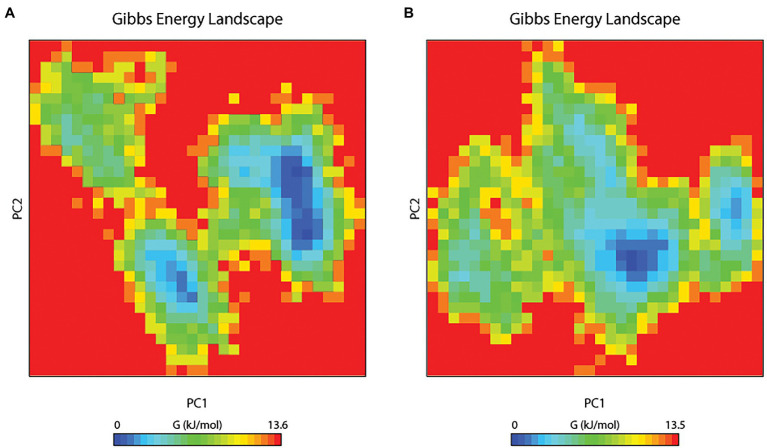
The Gibbs free energy landscape for protein–ligand complexes: PsoR-saponarin **(A)**, and PsoR-BOA **(B)**.

The principal component analysis (PCA) study helps in examining the important coordinated movements that occur during ligand binding. Only the first few eigenvectors are known to define the overall motion of the protein. In this study, the eigenvectors are obtained by diagonalizing the matrix. This study used the top 40 eigenvectors to estimate coordinated movements. Figure 8A shows the eigenvalues generated from the diagonalization of the covariance matrix of atomic fluctuations versus the appropriate eigenvector in decreasing order. Out of the 40 eigenvectors, the first 10 accounted for 88.15 and 91.16% of overall motions for BOA and saponarin, respectively. From PCA findings, this can be safely inferred that both of the studied complexes had lesser movements and form a stable association with PsoR. This could only be possible if the ligand association has altered protein structure and dynamics. Using PCA to construct 2D projection plots is another approach for obtaining the dynamics of protein–ligand complexes. Figure 8B depicts a two-dimensional projection of the trajectories in phase space for the first two main components, PC1, and PC2, for both complexes. In general, the complexes occupying a compact phase space along with a stable cluster are considered more stable, whereas those complexes taking greater space with unstable clusters are considered less stable. As they took up minimal area in the phase space, all of the complexes were found to be very stable. The above PCA and other MD simulation findings were also in accordance with the 2D PCA reports. Figure 9 shows the Gibbs energy landscape plot for PC1 and PC2 degrading complexes for saponarin and BOA. The results show that PsoR-saponarin and PsoR-BOA have a Gibbs free energy of 0–13.6 kJ mol^−1^ (Figure 9A) and 0–13.5 kJ mol^−1^ (Figure 9B), respectively. The free energy profiles of both protein–ligand complexes were significant, showing that the complexes remained stable at the time of the catalysis of saponarin and BOA. According to our studies, the PsoR complex was found to be thermodynamically efficient.

#### Binding Free Energy

The binding free energy was estimated from MD trajectories with the help of the MM-PBSA method executed in GROMACS. The overall binding energy of all protein–ligand complexes was found to be in the acceptable range. Precisely, complex PsoR-BOA showed the least binding free energy and greatest binding affinity with PsoR (−31.600 kJ mol^−1^), signifying a well-stabilized ligand conformation. For saponarin, the binding free energy was found to be −32.41KJmol^−1^ (Table 4). The results of the molecular docking and MD simulations were verified by the binding free energy calculation, confirming that these compounds predominantly bind to the PsoR. The MM-PBSA method has been utilized to quantify the exact binding free energy in biological reaction analysis. For the MM-PBSA study, the PsoR-saponarin and PsoR-BOA complexes were utilized, and the findings confirmed the predominant role played by PsoR protein in recognizing and binding further to at least two root exudates from the Poaceae family. We conclude that PsoR-saponarin and PsoR-BOA are stable complexes with remarkable binding affinities based on the overall MD Simulation (containing RMSD, RMSF, and RG analysis), post-MD simulation (including hydrogen bonds, SASA, gap, and PCA analysis), and binding free energy analysis findings.

**Table 4:** Table representing the Van der Waals, electrostatic, polar salvation, SASA, and binding energy for protein–ligand complexes.

Protein–ligand complex	Van der Waals energy (kJ mol^−1^)	Electrostatic energy (kJ mol^−1^)	Polar solvation energy (kJ mol^−1^)	SASA energy (kJ mol^−1^)	Binding energy (kJ mol^−1^)
PsoR-2-benzoxazolinone	−84.995 ± 7.863	−14.054 ± 13.469	77.036 ± 17.254	−9.586 ± 0.558	−31.600 ± 8.779
PsoR-saponarin	−141.523 ± 21.995	−61.966 ± 31.854	191.573 ± 44.689	−20.503 ± 2.115	−32.418 ± 19.219

## Discussion

Shortly after the discovery of LuxR solo proteins (LuxR orphan protein), it was realized that it interacts with some low molecular weight compounds from root exudates of the host plant and thus enables chemical communication between them, opening a new avenue toward the inter-kingdom signaling (González et al., 2013). PsoR is a LuxR solo regulator majorly present in plant-beneficial *Pseudomonas* spp. Interestingly, the presence of multiple PsoR orthologs in the genetic pool of plant-associated bacteria signifies both its widespread nature and possible role in mediating plant-bacterial signaling (Subramoni and Venturi, 2009). The studies on PsoR action have been limited as the crystal 3D structure was not available in the PDB databank. In this context, we have built a 3D model of PsoR for the first time, by using homology modeling. The modeled protein was subjected to energy minimization using the Swiss-PDB Viewer program. Further, to authenticate the accurateness of the predicted structure its ProQ and ProSA-web servers were employed. ProSA estimated *Z*-score value, −7.56 evincing highly reliable structure. ProQ generated LG and MaxSub scores of 9.991 and −0.605.

The Ramachandran plot created by the PDBsum server shows details on the arrangement of amino acid residues in the backbone dihedral angles Phi against Psi in the protein structure. Ramachandran dihedral statistics for modeled 3D structure of PsoR revealed a total of 92.8% residues in permitted regions. The results of the RC plot indicated that the built model had great geometry and was precise and reliable for future docking and MD simulations. Earlier studies suggested that the PsoR is likely specific for a molecule present in Poaceae plants. Therefore, to uncover a possible PsoR elicitor, we conducted a literature search and created a phytochemical library comprising 59 root exudates from barley, rice, rye, maize, and wheat plants. The molecular docking of the modeled PsoR, from AutoDock Vina, resulted in the best five compounds with lower or comparable binding energy to the reference molecule. As only two root exudates, i.e., saponarin and 2-benzoxazolinone (BOA), showed suitable binding with PsoR protein in MD simulations, we have elaborately discussed only these two exudates, i.e., saponarin and 2-benzoxazolinone (BOA) in our study. According to the LigPlot data, both these exudates can establish hydrogen bonds on the active site of the protein, implying that they could activate the enzyme. The reference molecule formed two hydrogen bonds with Ser42 and Tyr76. In our results, saponarin formed five hydrogen bonds with Pro43, Phe46, Ala49, His77, and Gln82, whereas BOA formed only one hydrogen bond with Ser141 and predominantly made hydrophobic interactions with Val38, Asp40, Tyr76, Asp79, Val81, Gln82, His121, Thr129, and Thr143. Both exudates displayed negative binding energy, making hydrogen and hydrophobic associations with active sites, suggesting a better association with PsoR. The more negative binding energy implied a relatively stable protein–ligand combination. In this study, both the complexes had fewer variations and lower RMSD during 100 ns, pointing that the PsoR enzyme can interact with both the exudates. During the interaction of PsoR with saponarin and BOA, the overall fluctuation recorded was between 0.4 and 0.2 as per the RMSD value; therefore, both exudates remained stable throughout MD simulations. The RG value of both exudates remained steady and got completely superimposed over PsoR protein. The increase in SASA value suggests that after encounter of PsoR with BOA and saponarin has led to a gradual unfolding of the critical residues in the active sites of PsoR protein. This contributes to stable binding of the small molecules to the protein. The PCA analysis identified the top 40 eigenvectors, with the first 10 constituting 88.15% of overall BOA movements, and 91.16% of total saponarin movements, which is only conceivable if the ligand interaction has altered protein structure and kinetics, suggesting that binding of saponarin and BOA, caused changes in the structure of PsoR.

Other recent studies have also utilized PCA to investigate protein–ligand complexes and found that every protein–ligand complex corresponds to a variable value of Gibbs free energy (David and Jacobs, 2014). In conclusion, MD simulation of the related protein–ligand complexes might help characterize the interactions of many additional exudate chemicals with PGPR proteins. Molecular dynamics study concludes that these two compounds are potent novel small molecules that might be used as lead molecules for the elicitation of PsoR protein to manifest its role in biocontrol by establishing inter-kingdom signaling. Saponarin is a natural diglycoside flavone (apigenin6-C-glucosyl-7-O-glucoside) compound present in the barley leaves. Similarly, 2-benzoxazolinone (BOA) is the degradation product of DIBOA (2,4-dihydroxy-2H-1,4-benzoxazin-3(4H)-one), one of the most abundant benzoxazinoids (BXs; Fomsgaard et al., 2006). It is produced in the growing root and shoots tissues; where after the addition of sugar moieties it is either retained in vacuoles or released from the roots. Saponarin is the major flavonoid found in barley, while BOA is the major secondary metabolite of wheat, maize, and rye. Earlier, González et al. (2013) concluded that PsoR protein most probably binds with root exudates compounds from the Poaceae family. Our findings are in line with this study, as both our root exudates compounds fall under the family Poaceae (González et al., 2013). Likewise, Neal et al. (2012) concluded that BXs are especially important in the rhizoplane of maize plants, where they attract the plant-friendly bacterium *Pseudomonas putida*. Recently, it has also been speculated that BXs increase the plant’s fitness by generating key metabolites, capable of inter-kingdom signaling (Schandry and Becker, 2020). In our study, we also found that 2-BOA binds effectively with the PsoR protein of *P. protegens* Pf5 and hence it is highly likely of showing cross-kingdom interaction. Based on the molecular docking and molecular dynamic results, we suggest that saponarin and BOA, the two root exudates of Poaceae, may have a potential eliciting effect in PsoR protein of plant-beneficial *Pseudomonas* spp.

## Conclusion

In this study, we have first-ever predicted the molecular interaction between the PsoR of *P. protegens* Pf5 and the root exudates of barley and wheat. For future research, it will be significant to look for *in vitro* experimental data to further decipher the entire metabolic pathway triggering the activation of biocontrol effects of the PsoR protein of plant-beneficial *Pseudomonas* spp.

## Data Availability Statement

All relevant data is contained within the article. The original contributions presented in the study are included in the article/Supplementary Material, further inquiries can be directed to the corresponding authors.

## Author Contributions

DS was the one who came up with the idea, designed the experiment, and wrote the first manuscript. The experiments were carried out by TJ and SP, who also evaluated the data and wrote the manuscript. VP and SM assisted with the manuscript’s writing and editing. MS and SC oversaw the research, assisted with data analysis, and wrote, reviewed, and edited the paper. All authors contributed to the article and approved the submitted version.

## Conflict of Interest

The authors declare that the research was conducted in the absence of any commercial or financial relationships that could be construed as a potential conflict of interest.

## Publisher’s Note

All claims expressed in this article are solely those of the authors and do not necessarily represent those of their affiliated organizations, or those of the publisher, the editors and the reviewers. Any product that may be evaluated in this article, or claim that may be made by its manufacturer, is not guaranteed or endorsed by the publisher.

## Footnotes

^1^ https://pubchem.ncbi.nlm.nih.gov
