# Supplementary material for: Identification of Putative Elicitors From Plant Root Exudates Responsible for PsoR Activation in Plant-Beneficial Pseudomonas spp. by Docking and Molecular Dynamics Simulation Approaches to Decipher Plant–Microbe Interaction
Source: Front Plant Sci. 2022 Apr 6;13:875494. doi: 10.3389/fpls.2022.875494 (PMC9020294; doi:10.3389/fpls.2022.875494)
Supplement: Supplementary file 1 [file Table_1.docx]

| **S.no.** | **PubChem ID** | **Compound Name** | **Binding Energy** |
| --- | --- | --- | --- |
| 1. | 637540 | o-coumaric acid | -6.6 |
| 2. | 6043 | 2-benzoxazolinone | -6.4 |
| 3. | 643757 | Cis-aconitic acid | -4.8 |
| 4. | 60961 | Adenosine | -6.2 |
| 5. | 196 | Adipic acid | -5.1 |
| 6. | 602 | Alanine | -4.0 |
| 7. | 439195 | Arabinose | -5.1 |
| 8. | 6322 | Arginine | -5.0 |
| 9. | 6267 | Asparagine | -5.2 |
| 10. | 5960 | Aspartic acid | -4.8 |
| 11. | 243 | Benzoic acid | -5.7 |
| 12. | 247 | Glycine Betaine | -3.6 |
| 13. | 689042 | Caffeic acid | -5.5 |
| 14. | 439224 | Carnosine | -5.3 |
| 15. | 305 | Choline | -3.6 |
| 16. | 1081 | Citramalic acid | -5.6 |
| 17. | 311 | Citric acid | -5.1 |
| 18. | 9750 | Citrulline | -5.0 |
| 19. | 586 | Creatine | -4.9 |
| 20. | 67678 | Cystine | -4.9 |
| 21. | 5793 | Glucose | -5.1 |
| 22. | 18950 | Mannose | -4.9 |
| 23. | 440667 | Dehydroascorbic acid | -5.1 |
| 24. | 752 | Glyceric acid | -4.2 |
| 25. | 6255 | D-Maltose | -5.5 |
| 26. | 11850 | Galactitol | -5.4 |
| 27. | 135191 | Xylose | -5.1 |
| 28. | 445858 | Ferulic acid | -5.6 |
| 29. | 2723872 | Fructose | -4.8 |
| 30. | 444972 | Fumaric acid | -4.4 |
| 31. | 119 | Gamma amino butyric acid (GABA) | -4.3 |
| 32. | 6036 | Galactose | -4.7 |
| 33. | 3469 | Benzoic acid | -5.1 |
| 34. | 10690 | Gluconic acid | -4.7 |
| 35. | 94715 | Glucuronic acid | -5.0 |
| 36. | 33032 | Glutamic acid | -5.0 |
| 37. | 5961 | Glutamine | -5.0 |
| 38. | 743 | Glutaric acid | -5.0 |
| 39. | 750 | Glycine | -3.5 |
| 40. | 760 | Glyoxylic acid | -3.3 |
| 41. | 135398634 | Guanine | -5.8 |
| 42. | 6274 | Histidine | -5.0 |
| 43. | 5810 | Hydroxyproline | -5.0 |
| 44. | 6306 | Isoleucine | -5.1 |
| 45. | 6106 | Leucine | -4.7 |
| 46. | 135 | p-hydroxybenzoic acid | -6.3 |
| 47. | 5962 | Lysine | -4.4 |
| 48. | 525 | Malic acid | -5.2 |
| 49. | 444539 | trans-cinnamic acid | -6.4 |
| 50. | 6137 | Methionine | -3.6 |
| 51. | 971 | Oxalic acid | -3.9 |
| 52. | 970 | Oxaloacetic acid | -5.2 |
| 53. | 10742 | Syringic acid | -5.1 |
| 54. | 18640396 | Phenylalanine | -4.8 |
| 55. | 1017 | Phthalic acid | -5.8 |
| 56. | 8468 | Vanillic acid | -5.5 |
| 57. | 145742 | Proline | -4.8 |
| 58. | 338 | Salicylic acid | -5.7 |
| 59. | 441381 | Saponarin | -7.1 |

**Supplementary Table 1.** A list of all the 59 docked root exudates from poaceae with their name, PubChem ID and binding energy.
